# An Open-Label, Single-Arm, Multicenter, Prospective, Post-Marketing Study to Evaluate the Effectiveness and Safety of Fluticasone Furoate/Vilanterol (FF/VI) Dry Powder Inhaler (DPI) in the Management of Asthma Patients (PROMISE-OD)

**DOI:** 10.7759/cureus.104295

**Published:** 2026-02-26

**Authors:** Ankit Kumar, Sandeepkumar Gupta, Punit K Jhawar, Suresh Raparthy, Satyajeet Sahoo, Sunil Kumar Raghumanda, Divya Bhojwani, Sagar Bhagat, Sumit Bhushan, Saiprasad Patil, Hanmant Barkate

**Affiliations:** 1 Respiratory Medicine, King George's Medical University, Lucknow, IND; 2 Internal Medicine, MV Hospital Research Centre, Lucknow, IND; 3 Internal Medicine, Dr Gillurkar Multispecialist Hospital, Nagpur, IND; 4 Pulmonology, St. Ann’s General and Cancer Hospital, Telangana, IND; 5 Internal Medicine, Utkal Hospital, Bhubaneshwar, IND; 6 Pulmonary Medicine, Andhra Medical College, Vishakhapatnam, IND; 7 Global Medical Affairs, Glenmark Pharmaceuticals Pvt. Ltd., Mumbai, IND

**Keywords:** asthma, dpi, fluticasone furoate/vilanterol, sabas, ultra labas

## Abstract

Background: A combination of inhaled corticosteroids (ICS) and long-acting β₂-agonists (LABA) is recommended for symptomatic asthma patients. Asthma control with single maintenance and reliever therapy (SMART) in patients remains poor with frequent nighttime awakenings, increased reliever use & severe exacerbations, including non-adherence. Once-daily FF/VI, an ultra LABA/ICS, has proven efficacy and safety in asthma with better adherence than multi-day regimens.

Methods: This prospective, multi-center, post-marketing (PROMISE-OD) study evaluated FF/VI DPI (100/25 mcg or 200/25 mcg) over 12 weeks in symptomatic asthma patients (≥12 years) on conventional therapies.

Results: Among 178 enrolled patients, 177 completed the study (53.9% females & mean age 44.8±15 years). Mean trough FEV1 improved by 180.5±810.7 ml (p=0.003) at week 4 & by 258.8±846.6 ml (p<0.0001) at week 12. The mean ACQ-5 score also reduced by -0.8±0.8 at week 4 and -1.3±0.9 at week 12 (p<0.0001). Adverse events occurred in 15.7% of patients, with no SAEs reported. Rescue medication was low (3.4% at week 4 & 5.1% at week 12). High patient and physician satisfaction was also observed.

Conclusion: FF/VI DPI demonstrated significant improvements in lung function and asthma control with high patient satisfaction and a favorable safety profile, establishing it as a promising maintenance therapy for asthma management in Indian patients.

## Introduction

Asthma is one of the most prevalent chronic non-communicable diseases, affecting over 260 million individuals worldwide and leading to more than 450,000 deaths annually [1]. In India, the condition affects approximately 35 million people, a significant portion of its 1.36 billion population [2]. The burden of asthma in India is substantial, representing 13.09% of the global asthma burden [3,4].

The Global Initiative for Asthma (GINA 2024) guidelines advocate ICS-formoterol single maintenance and reliever therapy (SMART) as the preferred approach for symptom relief at all stepwise asthma management algorithms [5]. However, despite this therapy, asthma may remain uncontrolled due to various factors. Potential contributing factors include suboptimal management, such as misdiagnosis and failure to address co-morbidities, improper inhaler technique, and poor treatment adherence [5]. Given these emerging concerns of uncontrolled asthma, a study conducted by Bateman et al. also reported that asthma control is seldom achieved by the SMART strategy. Among patients receiving SMART treatment, only 17.4% were deemed controlled, while 38.3% were classified as partly controlled, and 44.3% fell into the uncontrolled category. Also, it was observed that one in seven patients treated with SMART experienced severe exacerbation within the first year of therapy initiation [6].

Once-daily (OD) controller inhalers have been shown in previous studies to significantly improve treatment adherence compared to twice-daily (BID) controller medications, along with improvements in lung function and asthma symptoms [7]. Fluticasone furoate/vilanterol (FF/VI) is an ICS/ultra-LABA combination therapy approved for once-daily treatment of asthma in adults and adolescents aged ≥12 years in Europe and India and adults aged ≥18 years in the United States [8,9]. Vilanterol (VI) is a potent, selective β2-adrenoreceptor agonist with a long duration of action and rapid onset of action, demonstrating greater potency and higher intrinsic activity [10]. Fluticasone furoate (FF) has the highest relative GR binding affinity and negligible oral bioavailability with enhanced potency to reduce airway hyper-responsiveness with a wider therapeutic index (TI) compared with fluticasone propionate or budesonide [11].

Assessing the effectiveness and safety of the FF/VI dry powder inhaler (DPI) in a real-world Indian clinical setting would provide valuable insights for its further characterization. This post-marketing surveillance study was the first to evaluate the effectiveness and safety of the FF/VI DPI in Indian patients uncontrolled on conventional therapies, providing valuable insights into its role in asthma management.

## Materials and methods

This was a 12-week, multicentric, prospective, open-label, single-arm, post-marketing study in patients with asthma uncontrolled on conventional therapies. The study was conducted at six centers across India. The study enrolled patients ≥ 12 years of age with a clinical diagnosis of asthma as per GINA 2022 [12], having pre-bronchodilator FEV1 between 40-90% of predicted normal. Participants were prescribed FF/VI DPI by their treating physicians, and no interventions other than standard clinical practice were allowed.

Other inclusion criteria included patients who were symptomatic despite receiving ongoing conventional treatments (ICS-LABA/ICS-SABA/SMART) and had an ACQ-5 score ≥1.5 at baseline. Patients were excluded if they had a smoking history of more than 10 pack-years, were hospitalized with a life-threatening condition, had acute asthma exacerbation, or had hypersensitivity to any study drug components. The subjects were instructed to visit the clinic as part of routine clinical follow-up visits, preferably at 4 weeks (visit 2), 12 weeks (visit 3), or at any time if any adverse event occurs.

The detailed medical examination (vitals and physical examination) and ACQ-5 questionnaire were recorded at baseline and at weeks 4 & 12. Safety assessment was done throughout the study duration. Spirometry was also performed at all study visits by a trained technician. Subjects were provided with a subject diary to capture adverse events, rescue medications, and compliance with study medication.

The study adhered to the New Drugs and Clinical Trials Rules, 2019, issued by the Government of India [13], and followed ethical principles from the Declaration of Helsinki (64th WMA General Assembly, Fortaleza, Brazil, October 2013) [14] and ICH-GCP guidelines [15]. Ethical Committee (EC) approvals (approval numbers: SAIEC/11/2023/02; ECR/262/Inst/UP/2013/RR-19; IEC/12/03/23; GPL/CT/2022/011/IV; ECR/197/Inst/KGH/2013/RR-20; GPL/CT/2022/011/IV) were obtained from St. Ann’s Institutional Ethics Committee (SAIEC), Warangal; Institutional Ethics Committee, King George’s Medical University (KGMU), Lucknow; Institutional Ethics Committee, M.V. Hospital and Research Centre, Lucknow; Gillurkar Hospital Ethics Committee, Nagpur; Institutional Ethics Committee, King George Hospital, Visakhapatnam; and Centurion University of Technology and Management Independent Ethics Committee (CUTM-IEC), Utkal Hospital Bhubaneswar, Odisha prior to enrollment. Written informed consent or assent was obtained from all participants before study participation. The study was registered with CTRI under registration number CTRI/2023/10/058653, dated 13-Oct-2023. 

Effectiveness and safety assessment

This study evaluated the effectiveness through the mean change in trough FEV1, along with the mean change in ACQ-5 scores from baseline up to week 12, rescue medication use over 12 weeks, compliance with the study medication, and patient and physician satisfaction with the treatment. Other effectiveness variables included mean change in trough FVC and frequency of asthma exacerbations. Patient diaries were used to track the use of rescue medication and compliance with the study medication. The other endpoint was to evaluate the safety of FF/VI DPI in the treatment of patients with asthma in India in terms of the occurrence of treatment-emergent adverse events (TEAEs), serious TEAEs, and drug-related TEAEs.

Statistical analysis

All the data were summarized using descriptive statistics for continuous variables, including the number of patients, mean, standard deviation (SD), minimum, median, and maximum. For categorical variables, frequency and percentage were reported. The endpoint analyses of effectiveness and safety were conducted using the safety analysis set (SAF), full analysis set (FAS), and per protocol set (PPS) populations. The SAF included all subjects who received at least one dose of study drug treatment. The safety set was used for all safety analyses. The FAS population included all patients who received at least one dose of the study drug and had at least one post-baseline effectiveness assessment of FEV1. The PPS consisted of patients in the FAS without any major protocol deviations.

## Results

Patient demographics and clinical characteristics

During the study period, a total of 178 patients were screened and enrolled. Of these, 177 patients completed the study as PPS and 178 in SAF. The mean age of 178 participants was 44.8±15.0 years. The study had a relatively balanced gender distribution, with 96 (53.9%) female and 82 (46.1%) male participants. Among the enrolled patients, 20 (11.2%) had hypertension, 11 (6.2%) had diabetes mellitus, 3 (1.7%) had gastroesophageal reflux disease (GERD), 1 (0.6%) had allergic rhinitis, and 1 (0.6%) had hypothyroidism as comorbidities.

At baseline, the mean ACQ-5 score was 2.5±0.5, the mean trough FEV1 was 1493.7±757.9 ml ([65.2%±13.5] of predicted), and the mean FVC was 1839.1±750.5 ml. The majority of patients were on ICS/LABA 173 (97.19%) therapy at baseline, with formoterol/budesonide (FOR/BUD) SMART 114 (64.04%) as the most common.

Effectiveness

Improvement in Lung Function

**Figure 1:**
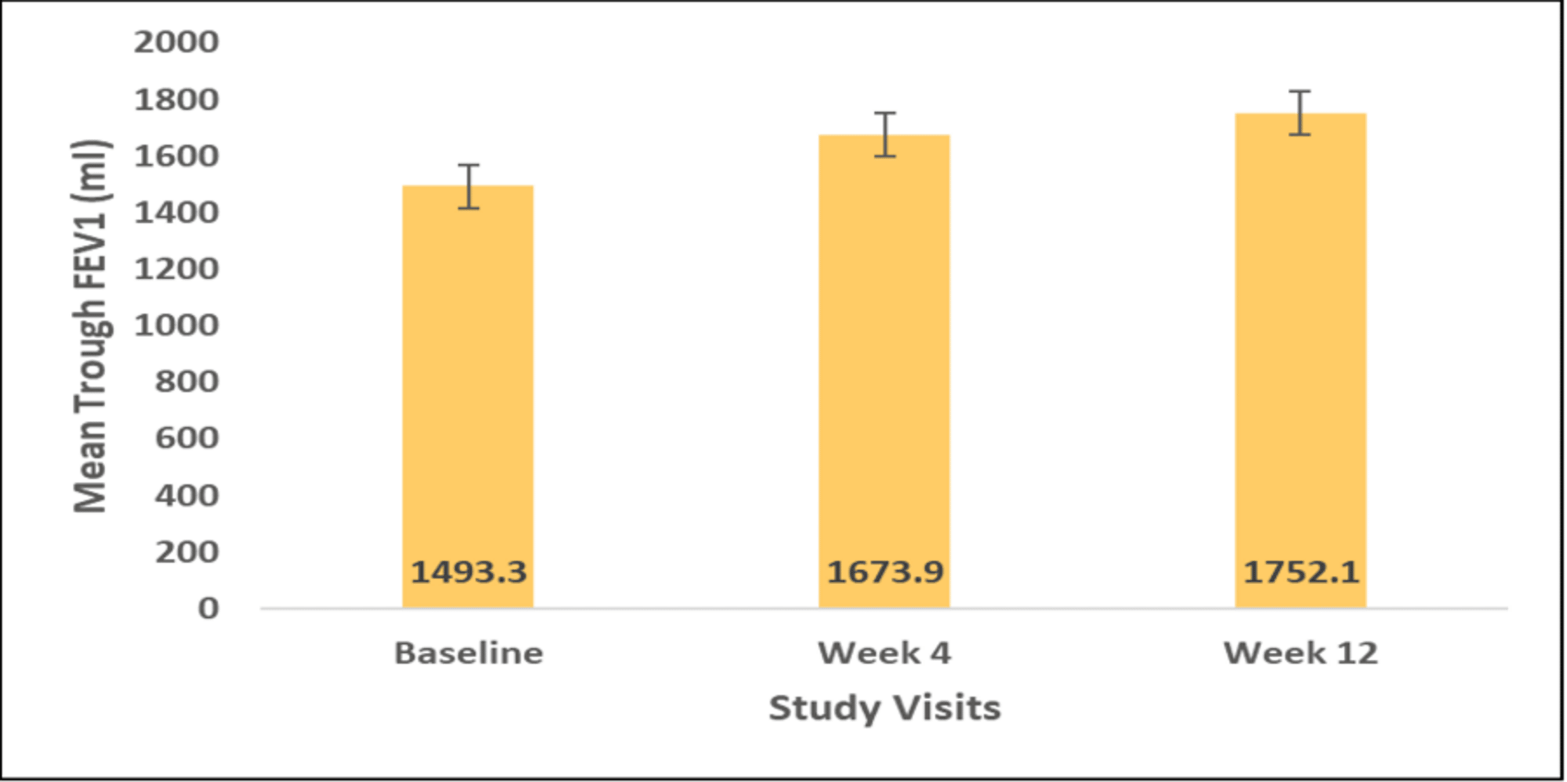
Mean trough FEV1 (ml) baseline, weeks 4 and 12 FEV1: Forced expiratory volume in one second

**Figure 2:**
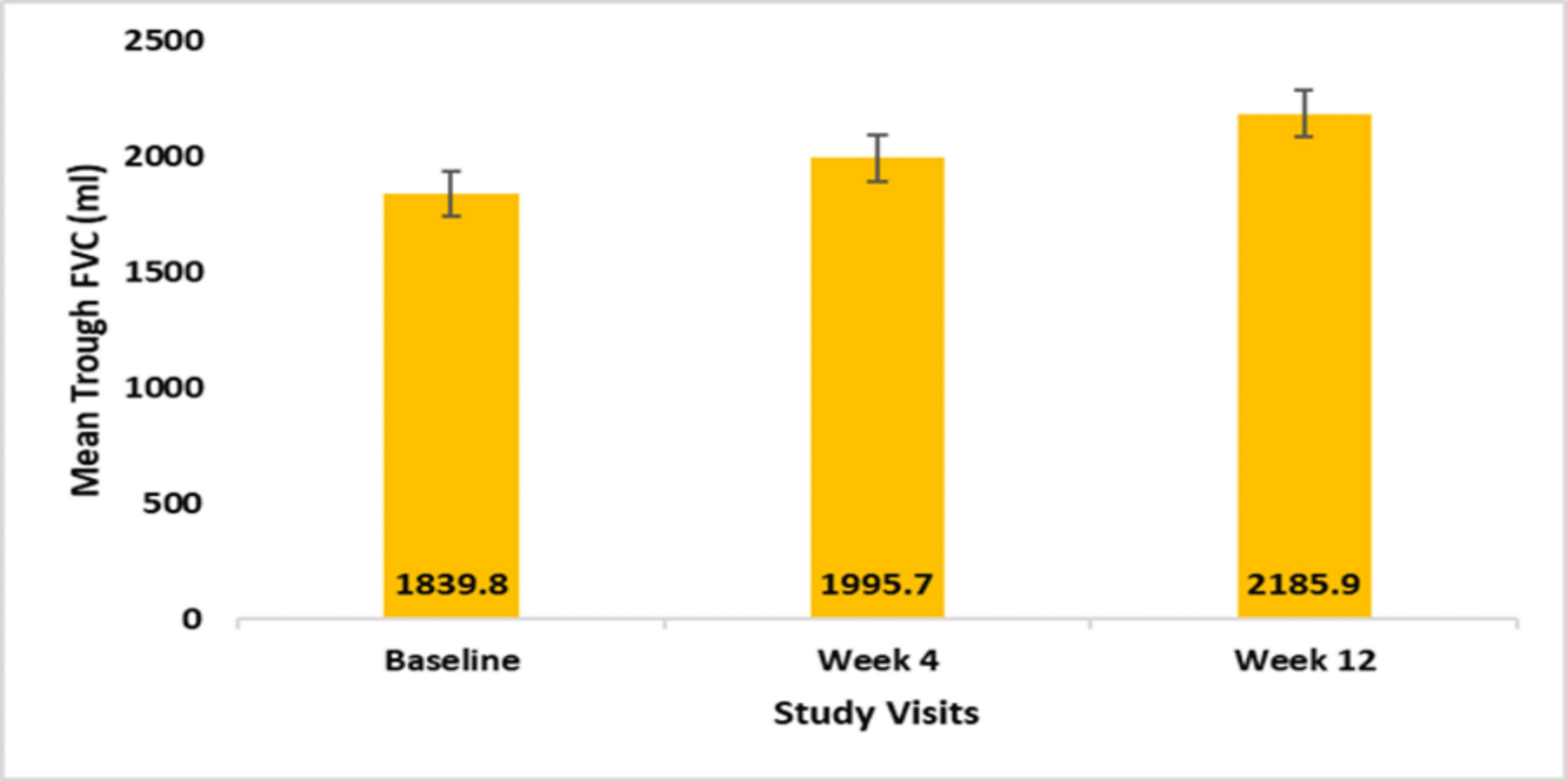
Mean trough FVC (ml) at baseline, weeks 4 and 12 FVC: Forced vital capacity

The FF/VI DPI (100/25 mcg or 200/25 mcg) produced statistically significant improvements in mean trough FEV1 and FVC from baseline to week 4 and by the end of the study visit. At baseline, the mean trough FEV1 was 1493.3 ± 760.1 ml, which increased to 1673.9 ± 539.8 ml at week 4 (Δ 180.5 ± 810.7 ml, p=0.0035) and 1752.1 ± 559.1 ml (Δ 258.8 ± 846.6 ml) at week 12 (p=0.0001) (Figure 1). Significant improvement was also observed in mean FVC at weeks 4 and 12. The mean trough FVC significantly improved from 1839.8 ± 752.6 ml at baseline to 1995.7 ± 715.2 ml at week 4 (Δ 155.9 ± 633.3 ml; p=0.0012) and to 2185.9 ± 716.5 ml at week 12 (Δ 346.0 ± 775.8 ml; p<0.0001) (Figure 2).

Improvement in Asthma Symptom Control

**Figure 3:**
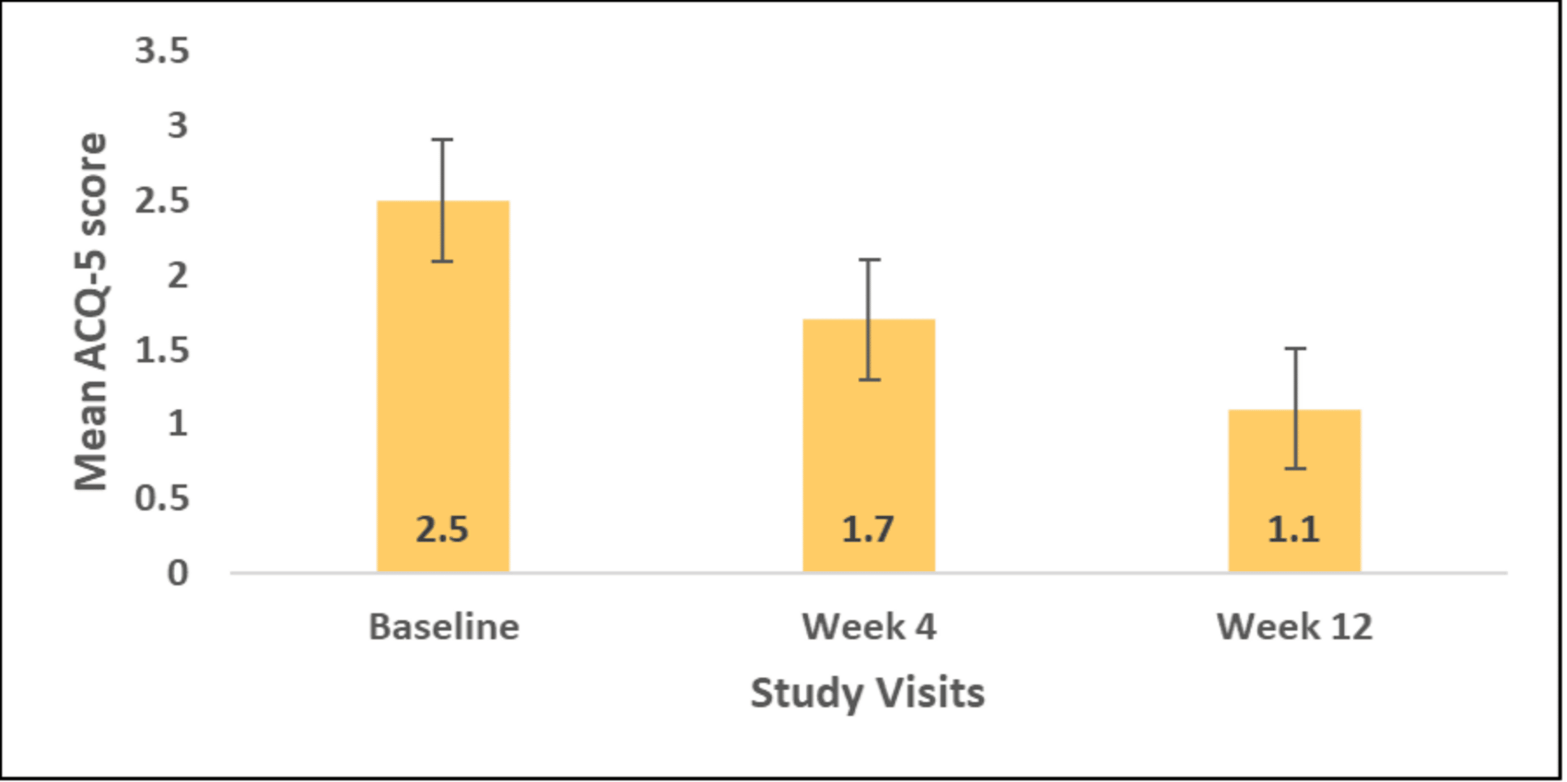
Mean ACQ-5 score at baseline, weeks 4 and 12 ACQ-5: Asthma control questionnaire. Baseline ACQ-5 score ≥ 1.5 for eligible patients [16].

The ACQ-5 score demonstrated continued and sustained improvement throughout the study period. At baseline, all the patients (100%) had poorly controlled asthma with a mean ACQ-5 score of 2.5 ± 0.5. With FF/VI DPI treatment, the mean ACQ-5 score significantly reduced to 1.7 ± 0.7 at week 4 (mean change: -0.8; p<0.0001) and to 1.1 ± 0.7 at week 12 (mean change: -1.3; p< 0.0001) (Figure 3).

Exacerbations

At baseline, almost half of the enrolled patients, 94 (53.10%), reported a history of exacerbation in the past year. Out of this, 62 (35.02%) of subjects had reported a history of ≥ 2 exacerbations. However, in this study, during 12 weeks of treatment with FF/VI DPI, only a small number of patients experienced exacerbations. At week 4, six patients (3.4%) reported mild exacerbations. By week 12, eight patients (4.49%) experienced mild exacerbations, and one patient (0.56%) had a moderate exacerbation. Notably, no severe exacerbations were observed during the entire study period.

Use of Rescue Medication

The overall use of rescue medication (salbutamol) remained low throughout the 12-week treatment period. At week 4, there were six patients (3.4%) who required rescue medication and nine (5.1%) by week 12.

Assessment of Patient’s & Physician’s Satisfaction

By the end of the study, the majority of the patients were very satisfied with FF/VI DPI treatment. Out of 177 patients, 167 patients (94.4%) found it easy to integrate FF/VI DPI into daily life, and 172 (97.2%) were confident that their asthma symptoms were controlled on current treatment, 167 (94.4%) found it easy to carry the inhaler without any complaint of taste and smell, and 171 (96.6%) were confident about the FF/VI DPI treatment. According to the treating physicians, all the patients at week 12 found it convenient to carry asthma inhalers with them and had no taste or smell complaints. Also, physicians found that all patients at week 12 were confident in using the inhaler effectively.

Compliance

All patients on FF/VI DPI reported adherence to the study medication during the entire study period, indicating a compliance rate of 100%.

Safety

Out of 178 patients in the safety population, a total of 22 (12.26%) patients experienced 28 adverse events (AEs), as summarized in Table 1. Most of the adverse events reported were mild (27, 96.4%), and only one (3.6%) was moderate. Amongst the total population, AEs reported in six (3.37%) patients were considered probable/likely due to the treatment, while AEs in 16 (8.99%) were considered unlikely by the investigator. Medication was required for adverse events in 18 patients (10.11%), while four patients (2.24%) experienced resolution without the need for medication. No serious adverse events were reported during the study, and all adverse events reported resolved completely with no study discontinuation.

**Table 1:** Summary of adverse events AE: Adverse Event, SAE: Serious Adverse Event, TEAE: Treatment-Emergent Adverse Event, n: Number of subjects, E: Number of Events

Adverse events	Number of adverse events reported N (%)
All TEAE's	28 (15.7%)
Gastrointestinal disorders	
Diarrhoea	1 (0.56%)
General disorders	
Pyrexia	6 (3.37%)
Musculoskeletal & connective tissue disorders	
Back pain	1 (0.56%)
Nervous system disorders	
Headache	7 (3.93%)
Respiratory, thoracic and mediastinal disorders	
Cough	5 (2.8%)
Nasopharyngitis	7 (3.93%)
Sneezing	1 (0.56%)

## Discussion

In this study, with patients that remained uncontrolled on conventional therapies (other ICS/LABA, including SMART or ICS/SABA), significant improvements in lung function were demonstrated following a 12-week treatment with a once-daily FF/VI DPI (100/25 mcg or 200/25 mcg). Notable improvements were observed through FEV1 and trough FVC, with consistent enhancement throughout the study duration relative to baseline values. Furthermore, patients exhibited improved ACQ-5 scores, reflecting better asthma control upon transitioning from twice-daily conventional therapy, including SMART, to a once-daily ICS/ultra-LABA regimen. The requirement for rescue medication was minimal throughout the study period, further supporting the efficacy of this treatment approach. The study also confirmed the safety and tolerability of FF/VI in the Indian population, as the combination therapy was well tolerated, with no serious adverse events reported. These findings reinforce the potential benefits of a once-daily ICS/ultra-LABA regimen for achieving superior asthma control in patients inadequately managed on conventional therapies.

A survey conducted by Chapman et al. found that nearly half of physicians (44%) and patients (46%) prioritize asthma symptom control as their primary treatment goal [17]. The Salford Lung Study (SLS-Asthma), a 12-month real-world trial, showed greater improvements in Asthma Control Test (ACT) scores with FF/VI compared to standard ICS ± LABA therapy, with 71% of FF/VI patients achieving ACT responder status by week 24 vs. 56% with FP/Salm [18]. Our study aligns with these findings, showing significant improvements in asthma symptom control with ACQ-5 score reductions of 32% at week 4 and 56% at week 12. By week 4, 56.49% of patients achieved the minimal clinically important difference (MCID) in ACQ-5, increasing to 73.44% by week 12. This underscores the effectiveness of switching to FF/VI DPI in improving asthma symptoms and overall disease control in Indian patients.

Lung function improvement is a critical aspect of asthma management. In this study, FF/VI treatment led to a mean increase in trough FEV₁ of 180.5 mL at week 4 and 259 mL at week 12, reflecting significant bronchodilation. These results are in agreement with findings from a Phase III trial conducted in India, which reported FEV₁ improvements of 216.4 mL at week 4 and 287.4 mL at week 12 with FF/VI pMDI [19]. The CAPTAIN study, a pivotal Phase III trial, reported a more modest increase of 76 mL and 24 mL with FF/VI 200/25 mcg and 100/25 mcg, respectively, by week 24 [20]. This suggests that FF/VI may provide a more pronounced improvement in lung function in real-world settings, particularly in patients with moderate-to-severe asthma requiring escalation of therapy.

Exacerbations significantly impact asthma outcomes and remain a key marker of disease control. The CAPTAIN and SLS-Asthma studies demonstrated that once-daily FF/VI effectively reduces exacerbation frequency, a trend that was also observed in our study [18,20]. By the end of 12 weeks, only 0.56% of patients experienced moderate exacerbation, while 4.49% had mild exacerbations, and no severe exacerbations were reported. These results highlight the potential of FF/VI in reducing exacerbation risk in Indian patients and support its role in achieving better disease stability compared to conventional therapies.

Excessive reliance on short-acting beta-agonists (SABAs) is associated with an increased risk of exacerbation and mortality, as emphasized by GINA 2024 guidelines. Despite the growing adoption of SMART therapy, a survey found that 85% of patients on SMART therapy were still provided with a separate rescue inhaler, indicating potential gaps in real-world implementation [21]. In our study, rescue salbutamol inhaler use was minimal, with only 3.4% of patients requiring it at week 4 and 5.1% at week 12, demonstrating that FF/VI provided adequate symptom control, reducing the need for additional bronchodilation.

Adherence is a well-recognized challenge in asthma management, with twice-daily (BID) regimens often leading to treatment discontinuation. Real-world studies have shown that once-daily FF/VI improves adherence rates and reduces the risk of therapy discontinuation compared to BID inhalers [22]. In our study, all patients remained compliant with FF/VI therapy at weeks 4 and 12, with no discontinuations reported, reinforcing the advantage of once-daily administration in enhancing treatment persistence and long-term disease control.

The safety profile of FF/VI has been well-established in both clinical trials and real-world settings [18,19,23]. A 12-week randomized study conducted across Europe and Asia comparing FF/VI (100/25 mcg) once daily to FP/Salm (500/50 mcg) twice daily found similar rates of adverse events in both groups, with headache and nasopharyngitis being the most commonly reported [18]. Our study findings were consistent with these data, with 15.7% of patients reporting adverse events, the majority being mild in nature (96.4%). The most frequently reported events were nasopharyngitis, headache, and pyrexia, and importantly, no serious adverse events (SAEs) were observed. These findings reaffirm the favorable safety profile of FF/VI in Indian patients, with no new safety signals identified.

A key strength of this study is that it provides the first real-world evidence on the effectiveness of FF/VI DPI in Indian patients previously uncontrolled on conventional therapies. The study demonstrated significant improvements in asthma control, lung function, adherence, and exacerbation reduction, aligning with global clinical trials. Additionally, this study assessed patient and physician satisfaction, a factor not previously reported. Findings revealed high satisfaction levels, with patients finding the inhaler easy to use and expressing confidence in symptom management, while physicians reported trust in its efficacy and ease of use. Importantly, concerns about medication use decreased over time, reinforcing the user-friendly nature of FF/VI DPI and its positive impact on adherence. However, certain limitations should be considered. The open-label, single-arm design lacks a comparator group, potentially introducing bias in treatment assessment. The 12-week follow-up limits the evaluation of long-term outcomes and disease progression. Additionally, the study primarily included moderate-to-severe asthma patients already on ICS/LABA or ICS/SABA, limiting applicability to milder asthma cases.

## Conclusions

In conclusion, consistent with the large-scale clinical studies and real-world evidence, our study shows that once-daily FF/VI DPI reduces airflow obstruction and improves symptoms and asthma control in Indian patients uncontrolled on conventional twice-daily therapies. Furthermore, no additional safety concerns have been found, indicating FF/VI has a positive benefit-to-risk profile and provides a valuable treatment option for Indian patients with inadequately controlled asthma.

## References

[1] (2026). Global Initiative for Asthma (GINA): World asthma day 2024. https://ginasthma.org/world-asthma-day-2024/.

[2] Global Asthma Network (2026). Global Asthma Network: The global asthma report 2025: patient stories. Global Asthma Report.

[3] GBD 2019 Diseases and Injuries Collaborators (2026). Institute for Health Metrics and Evaluation: GBD 2019 diseases and injuries collaborators: GBD compare data visualization. https://www.healthdata.org/data-tools-practices/interactive-visuals/gbd-compare.

[4] Singh S, Salvi S, Mangal DK (2022). Prevalence, time trends and treatment practices of asthma in India: the Global Asthma Network study. ERJ Open Res.

[5] (2026). Global Initiative for Asthma: Global strategy for asthma management and prevention. http://www.ginasthma.org.

[6] Imam SF, Zafar S, Oppenheimer JJ (2022). Single maintenance and reliever therapy in treatment of asthma exacerbations. Ann Allergy Asthma Immunol.

[7] Chapman KR (2010). SMART isn't. J Allergy Clin Immunol.

[8] De Keyser H, Vuong V, Kaye L (2023). Is once versus twice daily dosing better for adherence in asthma and chronic obstructive pulmonary disease?. J Allergy Clin Immunol Pract.

[9] Albertson TE, Bullick SW, Schivo M, Sutter ME (2016). Spotlight on fluticasone furoate/vilanterol trifenatate for the once-daily treatment of asthma: design, development and place in therapy. Drug Des Devel Ther.

[10] (2026). Central Drugs Standard Control Organization (CDSCO): Subject expert committee (SEC) proceedings. https://cdsco.gov.in/opencms/opencms/en/Committees/SEC/.

[11] Aparici M, Gavaldà A, Ramos I (2016). In vitro and in vivo preclinical profile of abediterol (LAS100977), an inhaled long-acting β2-adrenoceptor agonist, compared with indacaterol, olodaterol and vilanterol. Eur J Pharmacol.

[12] (2026). Global Imitative Asthma: 2022 GINA main report. https://ginasthma.org/gina-reports/.

[13] NEW DRUGS AND CLINICAL (2025). Central Drugs Standard Control Organization, Ministry of Health and Family Welfare, Govt. of India: New drugs and clinical trials rules. https://www.cdsco.gov.in/opencms/opencms/system/modules/CDSCO.WEB/elements/download_file_division.jsp?num_id=OTg4OA==.

[14] (2025). World Medical Association: WMA Declaration of Helsinki - ethical principles for medical research involving human participants. Adopted June.

[15] (2026). International Council for Harmonisation of Technical Requirements for Pharmaceuticals for Human Use (ICH): Guideline for good clinical practice. https://database.ich.org/sites/default/files/ICH_E6%28R3%29_Step4_FinalGuideline_2025_0106.pdf.

[16] Juniper EF, Bousquet J, Abetz L, Bateman ED (2006). Identifying 'well-controlled' and 'not well-controlled' asthma using the Asthma Control Questionnaire. Respir Med.

[17] Chapman KR, An L, Bosnic-Anticevich S (2021). Asthma patients' and physicians' perspectives on the burden and management of asthma. Respir Med.

[18] Jacques L, Bakerly ND, New JP (2019). Effectiveness of fluticasone furoate/vilanterol versus fluticasone propionate/salmeterol on asthma control in the Salford lung study. J Asthma.

[19] Kumar A, Jain MK, Barge VB (2024). Efficacy and safety of once-daily vilanterol/fluticasone furoate MDI in persistent asthma: phase 3 OD-INHALE study. J Asthma.

[20] Lee LA, Bailes Z, Barnes N (2021). Efficacy and safety of once-daily single-inhaler triple therapy (FF/UMEC/VI) versus FF/VI in patients with inadequately controlled asthma (CAPTAIN): a double-blind, randomised, phase 3A trial. Lancet Respir Med.

[21] Domingo C, Singh D (2023). The changing asthma management landscape and need for appropriate SABA prescription. Adv Ther.

[22] Averell CM, Stanford RH, Laliberté F (2021). Medication adherence in patients with asthma using once-daily versus twice-daily ICS/LABAs. J Asthma.

[23] Agustí A, de Teresa L, De Backer W (2014). A comparison of the efficacy and safety of once-daily fluticasone furoate/vilanterol with twice-daily fluticasone propionate/salmeterol in moderate to very severe COPD. Eur Respir J.

